# Medical Education and State Medical Examinations

**Published:** 1896-04

**Authors:** William Warren Potter

**Affiliations:** Buffalo, N. Y.; Member New York State Medical Examining and Licensing Board. President National Confederation State Medical Examining and Licensing Boards, 1896


### MEDICAL EDUCATION AND STATE MEDICAL EXAMINATIONS.

Conducted by WILLIAM WARREN POTTER, M. D., Buffalo, N. Y.

Member New York State Medical Examining and Licensing Board.

President National Confederation State Medical Examining and Licensing Boards, 1896.

### UNIVERSITY OF THE STATE OF NEW YORK MEDICAL EXAMINATIONS.

Examinations for license to practise medicine in this state will be held as follows :

*Dates.*—1896 : April 7-10, May 19–22, June 16–19.

*Places.*—New York, Albany, Syracuse, Buffalo. Each candidate is notified as to exact place.

*Daily Program.*—Tuesday morning, 9.15—12.15, anatomy ; afternoon, 1.15—4.15, physiology and hygiene. Wednesday morning, 9.15—12.15, chemistry ; afternoon, 1.15—4.15, surgery.

Thursday morning, 9.15—12.15, obstetrics ; afternoon, 1.15—4.15, pathology and diagnosis. Friday morning, 9.15—12.15, therapeutics.

---

### NATIONAL CONFEDERATION OF STATE MEDICAL EXAMINING AND LICENSING BOARDS.

THE executive council of the National Confederation of State Medical Examining and Licensing Boards has issued the following circular, which emanates from the office of the secretary, Dr. Benjamin M. Griffith, Springfield, Ill. :

The sixth annual meeting of this organisation will be held in room No. 1, Hotel Aragon, at Atlanta, Ga., Monday, May 4, 1896, at 10 o'clock A. M.

The following program will be carried out—namely :

1. Introductory remarks by the vice-president.
2. Report of the committee on revision of the constitution and by-laws.
3. Discussion and action thereon.
4. Report of the secretary.
5. Annual address of the president.
6. Address. Preliminary education, training and practice in New York. By James Russell Parsons, Jr., director of examinations, University of the State of New York, Albany.
7. Paper : Some obstacles to an inter-state recognition of a state license to practise medicine, with suggestions for their removal. By Charles McIntire, M. D., Easton, Pa.
8. Paper.———By Jos. M. Mathews, M. D., Louisville, Ky.
9. Paper.———By Wm. S. Foster, M. D., Pittsburg, Pa.
10. Miscellaneous business.
11. Election of officers.

The objects of the confederation, though purely of an advisory nature, are to discuss questions that pertain to state licensure in medicine with a view to a comparison and improvement of methods, a collection and dissemination of information on the subject, and to consider any and all propositions that have for their purpose the advancement of the standard of medical education in the United States.

The officers of the confederation, therefore, beg to extend a cordial invitation to members of state examining boards and all ex-members of state boards, as well as to every physician and educator who is friendly to the objects sought, to attend the meeting and participate in the proceedings.

### THE REGENTS' DEPARTMENT.

The annual report of the regents was made public in the legislature, February 11, 1896. It discusses at length preliminary and professional requirements for degrees. It reviews briefly the question of dental law and medical students' examinations, showing New York to be in the lead in all that tends to uniform standards in the professions. The report mentions the attempts made by other states to adopt regents' standards and the influence of the regents' registered list in the elevation of the character of the institutions seeking recognition. Credentials were received last year in twenty different languages from applicants for professional certificates. The consideration of these credentials involves a careful study of the educational system throughout the world. It is safe to say that the registry list of the Board of Regents of the University of the State of New York is the most complete in existence, furnishing information to inquiries from the most remote quarters of the globe.

It is easy to show that New York is the leading state in matters pertaining to higher medical education. Since 1892 there has been a growth of about 700 in the number of medical students and of more than 400 in the number of medical students from other states and countries who have come to study in New York institutions. There has been an increase of over 500 per cent. in the number of post-graduate students. The sum invested in medical schools increased from $2,764,530 in 1891 to $4,061,293 in 1895, and the amount of money expended for medical education increased from $365,405 in 1891 to $697,653 in 1895.

The recommendations of Secretary Dewey and his review of the work of the various departments had not been completed when the report was made public.

---

### METHODS OF MEDICAL TEACHING.

The American Academy of Medicine, which holds its annual meeting at Atlanta, May 2 and 4, 1896, announces a discussion on the above subject as follows:

I. *Preliminary.*—(1) The preparatory mental discipline for the medical student; (2) The subjects to be known before beginning the study of medicine, Dr. F. N. Gerrish, Portland, Me.

II. *Methods.*—(3) The lecture and its uses, Dr. C. B. Penrose, Philadelphia. (4) Text-book recitation and its advantages, Dr. DeLancey Rochester, Buffalo, N. Y. (5) Laboratory methods,

Dr. V. C. Vaughan, Ann Arbor. (6) Clinical instruction, Dr. J. Madison Taylor, Philadelphia. (7) The Seminar method, Dr. Bayard Holmes, Chicago. (8) Pass examinations ; (9) The final examination, Dr. E. L. Holmes, Chicago. (10) Students' medical societies, Dr. Roswell Park, Buffalo, N. Y. (11) State examination, Dr. J. McPherson Scott, of the Maryland board. (12) The best method to teach anatomy, Dr. J. B. Roberts, Philadelphia. (13) The best method to teach physiology, Dr. C. D. Smith, Portland, Me. (14) The best method to teach inner medicine, Dr. J. C. Wilson, Philadelphia. (15) The best method to teach surgery, Dr. J. S. Wight, Brooklyn. (16) The best method to teach obstetrics, Dr. J. C. Edgar, New York. (17) The best method to teach state medicine, Dr. George H. Rohé, Catonsville.

---

### MEDICAL TRAINING IN NEW YORK.

THE standard of medical education (*New York Tribune*) in this state is threatened with a serious danger. Through a series of years it has been the aim of the regents of the university, in coöperation with the leading physicians, to prevent the medical degree from being conferred in New York on any but men of high character and attainments. Their task has not been easy. Incompetent persons seek to gain admission to every profession, and schools are always to be found willing to further their designs. Every effort to sift more carefully applications for permission to practise medicine has been strenuously resisted, but finally the requirements for degrees have been made uniform and sufficiently strict to guarantee the fitness of the recipients for their work. It appears, however, that these regulations affect injuriously the interests of some students, who deem themselves none the less competent for general practice because they cannot technically comply with the law, and of some teachers, who believe they can educate physicians as good as any on somewhat easier terms than the statute permits. Accordingly there is now a bill before the legislature, introduced by Mr. Stanchfield, changing the requirements for medical examinations.

At the hearing before the judiciary committee of the assembly, leading doctors from all parts of the state and representatives from many of the medical colleges presented weighty objections to the Stanchfield bill, and the state medical society, through its committee on legislation, sent a protest. It was urged that there would be little objection to the four-year course proposed, if the enactment did not carry with it the practical repeal of present safeguards for the protection of people from improper treatment and open the door to abuses and the cheapening of the medical degree. The examinations under the present law are denounced as "arbitrary" by those who favor the Stanchfield bill, but arbitrary requirements in such a case are surely preferable to loose ones. The proposed measure orders examinations for matriculation in medical colleges and certain studies are named as essential, but no standard of perfection is prescribed, and every medical school is left free to matriculate students on its own terms, so long as it goes through the form of examination.

Exemption is also allowed to graduates or matriculates of "reputable colleges or high schools of the first grade," leaving it to the examining officer to determine for himself what colleges and schools are "reputable." The safeguards as to age, character and satisfactory instruction are left out of the law, and a medical school which so conducts itself as to retain its charter can graduate students without making them conform to any general standard of excellence. Moreover, the bill is so drawn that students have a year in which to matriculate under the present law, and thus secure exemption from the necessity of studying four years, and afterward may take advantage of the want of restrictions in the substitute measure against the invasion of the ranks of the medical profession by men of bad character and inadequate preparation.

The general welfare demands that no person shall be intrusted with the health and lives of others who has not proved, under the most rigid scrutiny, his competence for the task and the good faith in which he will perform it. The practitioner must keep pace with the progress of science and the medical degree must year by year come to stand for greater knowledge and ability. To secure this, severe rules must be enforced on all. Doubtless such rules cause hardship in some cases and keep out of the profession individuals who might practise it worthily. The honesty and ability of the management of particular schools which might desire a larger liberty may be conceded. But the fact must be faced that there are unprincipled persons ever ready to take advantage of a loophole in the law ; and while those who now favor the Stanchfield bill might conduct themselves under it with perfect propriety, there is no guarantee that others might not abuse the opportunities it would give them. The fact that students find it easier to get diplomas in other states is no valid ground for making degrees cheap here. The first duty of the State of New York is to make its medical degrees as good as any in the world. We have long been working in that direction. There should be no retrogression.

---

ADVICE TO THOSE WHO CONTEMPLATE THE STUDY OF MEDICINE.

The *British Medical Journal* for September 7, 1895, has some very wise words for young men who are considering the advisability of entering the medical profession. It is of the utmost importance, it says, that the student and his advisers should have a clear idea of the object to be aimed at. Life-long disappointment may be the consequence of a false step at the outset. Among the careers in which the highest prizes are open to all who have wit and energy and can afford the cost of the necessary course of study, medicine offers to many the highest attractions. The scientific character of the study, the purely personal nature of the work, the life of intimacy with many people of many ranks, the possibility—dim perhaps, but still the possibility,—of wealth and honor, and the almost certainty, at least, of bread and cheese as the reward of patience, sobriety and hard work, are sure to draw many to medicine as their career in life. Those who find their way to wealth, influence and position are few. To the majority who commence their professional studies in medicine in October, medicine will prove a harsh mother, and will give little beyond the necessaries of a simple and frugal life. The man who is to succeed must give himself up to being a student for five years at least, and that means that he must have sufficient capital to keep himself during that time as well as to pay the necessary fees. It is no small matter to fix one's life beforehand for a certain five or six years, and the importance of the decision is in no way lightened by the knowledge that a medical education is peculiarly special, leads to little else but medicine, is of no service in obtaining entry into any other profession or even trade, and that unless it can be carried through to the end it means so much loss of time.—*Canadian Medical Review.*

---

PENNSYLVANIA STATE BOARD OF MEDICAL EXAMINERS.

The State Board of Examiners for a license to practise medicine in Pennsylvania has passed over its first year (*Post-Graduate*), and according to Dr. Cranch, of Erie, a member of that board, "over 5 per cent. of all schools failed utterly, and many papers, even among those finally passed, showed a deplorable lack of general and special culture." In the opinion of the Pennsylvania examiners the new law has been fully justified. There is no doubt that this is the case in New York after some three or four years of experience.

Dr. Cranch also believes that through the laws creating medical examiners "we shall finally see the cessation of medical sectarianism by demonstrating," as he says, "the real unity of all schools on most subjects." These laws are accomplishing much, so that we may hope to see the day when the only name to a medical practitioner will be physician or surgeon.

---

THE Georgia state examining boards, in 1895, (*Atlanta Medical and Surgical Journal*,) examined and licensed 125 applicants ; 105 were regulars, thirteen eclectics and seven homeopaths. It is said to have been the policy of the boards not to be too severe in their examinations all at once. It is expected that they will be more rigid in the future. We hope that the boards will see that the spirit and letter of the law are fully carried out. Its friends look to it as the only means of protecting our people from hordes of medical tramps and numbers of incompetent graduates. The law is a good one and deserves to be carried out.

---

### OREGON STATE MEDICAL EXAMINING BOARD.

THE *California Medical Journal* has published a paper on the subject of the Oregon State Medical Examining Board, (*Medical Sentinel*, February, 1896,) written by Dr. McConnell, of the board, in which we note that during its first six months forty-three applicants have been examined. Of these, several have been obliged to take a second examination. Of the total, forty-three, examined, forty-two have passed the board. On July 2d, fourteen were examined and six failed, but on August 1st these six were reëxamined and all passed the board. Of those examined, thirty-seven were regulars, three eclectics and two homeopaths. The unfortunate individual who failed to pass was an "allopath." Dr. McConnell says:

In behalf of the new board, I may say that it is one of the many boards that cannot be bribed. At least, so far as I am aware, it has been able to withstand the offers of all comers thus far. Just how long we may be able to keep this up I am not authorised to state.

---

### OHIO STATE BOARD OF MEDICAL EXAMINATION AND REGISTRATION.

OHIO has at last been compelled, in pure self-defense, to pass a medical practice law, and we hope our good neighbor will soon be able to clear out the army of quacks and irregular practitioners that has made the Buckeye State a camping-ground for so many years. After struggling for several years with the legislature, the friends of reform in medical education finally triumphed, and what is known as the Kimmel medical bill became a law February 19, 1896. The following is a synopsis of the law:

The governor shall appoint seven medical men, constituting the "medical board," to serve one, two, three, four, five, six and seven years respectively, such board to be made up from the various so-called schools in proportion to their numerical strength, but no one "school" to have a majority of the board. A college graduate must present his diploma with his affidavit that he is lawfully possessed of the same, and give age and time spent in study. If the board finds the diploma genuine and from a legally chartered institute, he shall be granted a certificate, which must be filed with the probate judge. If a medical practitioner, not a graduate, furnish the board an attested affidavit, stating period and places where he practised, and such statement is satisfactory to the board, a certificate shall be granted. All other applicants for certificates, who are not legal practitioners under the laws, shall pass such examination as the board may require.

The Kimmel law also provides for the regulation of the practice of midwifery. All midwives must register with the probate judge of the county where they reside, giving age, education received, time of practice, and the like. Midwives shall have no right to perform version, treat breach or faee presentations or use instruments. The bill provides for fees, penalties, revocation of license, and the like. It is not an ideal law, but it is a beginning and we hope will lead to a better one in the near future.

---

### EFFECTS OF THE MEDICAL LAW IN RHODE ISLAND.

THE law regulating the practice of medicine in Rhode Island (*Atlantic Medical Weekly*, December 14, 1895,) has now been in operation some over three months and satisfactory results have followed. In all, 480 certificates have been issued, of which 436 were on first or diploma form and forty-four on second or time limit form. The grounds for refusal have been numerous and consist of unprofessional conduct, itinerancy, inability to present satisfactory evidence of having been engaged in practice in this state prior to 1892 and refusal to submit to examination.

Many physicians out of the state have applied for certificates. Some are young physicians who really intend to locate in Rhode Island until they are convinced that the field is not sufficiently lucrative to warrant their remaining. Others have applied with the idea that it would be to their advantage to have certificates from all states, so that their way would be clear if they should wish to change their field, and yet others have applied for the purpose of ascertaining whether they could practise or not before coming.

In order to prevent mistakes in issuing certificates to the latter class, and to prevent promiscuous distribution of them throughout the country, the board made a ruling that no certificate should be issued to an applicant, no matter what his credentials, until he had located and established himself here for a sufficient length of time to assure the board that he intended to practise here. This ruling has proved a necessary one, and has prevented many from registering from outside the state.

---

### SIX OUT OF NINE.

THE following list (*Medical Sentinel*) of those who passed the examination of the Washington State Board of Medical Examiners in January, 1896, is kindly furnished by Dr. W. W. Misner, secretary of the board: J. Janson, Seattle, University of Minnesota, 1892; Halfdan Slippern, Seattle, University of Minnesota, 1895; H. S. Goddard, Portland, Ore., Willamette University, 1888; M. L. Doom, Chehalis, Eclectic Medical College, 1874; Henry S. Strickland, Olympia, Physicians and Surgeons, New York, 1877; R. O. Ball, West Ferndale, Eclectic Medical Institute, Cincinnati. Three others failed to pass.

---

A CASE of tetanus is reported in the *Deutsche Med. Wochenschrift*, No. 36, in which the patient died in spite of antitoxin treatment. Eighteen injections of the serum were given. The patient had had trismus for nine days before the treatment was instituted.—*Polyclinic.*

